# Transient integral boundary layer method to calculate the translesional pressure drop and the fractional flow reserve in myocardial bridges

**DOI:** 10.1186/1475-925X-5-42

**Published:** 2006-06-21

**Authors:** Stefan Bernhard, Stefan Möhlenkamp, Andreas Tilgner

**Affiliations:** 1 Department of Physics, Georg-August-Universität Göttingen, Friedrich-Hundt-Platz 1, 37077 Göttingen, Germany; 2 University Clinic of Essen, West-German Heart Center, Clinic of Cardiology, Hufelandstrasse 55, 45122 Essen, Germany

## Abstract

**Background:**

The pressure drop – flow relations in myocardial bridges and the assessment of vascular heart disease via fractional flow reserve (FFR) have motivated many researchers the last decades. The aim of this study is to simulate several clinical conditions present in myocardial bridges to determine the flow reserve and consequently the clinical relevance of the disease. From a fluid mechanical point of view the pathophysiological situation in myocardial bridges involves fluid flow in a time dependent flow geometry, caused by contracting cardiac muscles overlying an intramural segment of the coronary artery. These flows mostly involve flow separation and secondary motions, which are difficult to calculate and analyse.

**Methods:**

Because a three dimensional simulation of the haemodynamic conditions in myocardial bridges in a network of coronary arteries is time-consuming, we present a boundary layer model for the calculation of the pressure drop and flow separation. The approach is based on the assumption that the flow can be sufficiently well described by the interaction of an inviscid core and a viscous boundary layer. Under the assumption that the idealised flow through a constriction is given by near-equilibrium velocity profiles of the Falkner-Skan-Cooke (FSC) family, the evolution of the boundary layer is obtained by the simultaneous solution of the Falkner-Skan equation and the transient von-Kármán integral momentum equation.

**Results:**

The model was used to investigate the relative importance of several physical parameters present in myocardial bridges. Results have been obtained for steady and unsteady flow through vessels with 0 – 85% diameter stenosis. We compare two clinical relevant cases of a myocardial bridge in the middle segment of the left anterior descending coronary artery (LAD). The pressure derived FFR of fixed and dynamic lesions has shown that the flow is less affected in the dynamic case, because the distal pressure partially recovers during re-opening of the vessel in diastole. We have further calculated the wall shear stress (WSS) distributions in addition to the location and length of the flow reversal zones in dependence on the severity of the disease.

**Conclusion:**

The described boundary layer method can be used to simulate frictional forces and wall shear stresses in the entrance region of vessels. Earlier models are supplemented by the viscous effects in a quasi three-dimensional vessel geometry with a prescribed wall motion. The results indicate that the translesional pressure drop and the mean FFR compares favourably to clinical findings in the literature. We have further shown that the mean FFR under the assumption of Hagen-Poiseuille flow is overestimated in developing flow conditions.

## Background

The incomplete understanding of the pathophysiology and clinical relevance of myocardial bridges has been the subject of debate for the last quarter century. An overview of physiological relevant mechanisms of myocardial bridging, the current diagnostic tools and treatment strategies are found in [1-8]. Despite extensive studies on this subject there is no consensus on its clinical significance to myocardial ischaemia or angina pectoris.

A variety of models concerned with arterial stenoses [9-12] and series of stenoses [13-15] are found in the literature. Theoretical studies have been done to predict the location of maximum wall shear stress [16-18] and the extent of flow separation located distal to fixed stenoses [19-21]. There are a few models, which discuss flow in a time dependent two-dimensional flow geometry [22-25]. These models assume rigid walls and are mainly focused on vortex formation and the wall shear stress distribution. However, [26] discussed the extent of the separation zone in a one-dimensional empirical parameter model using the concept of dividing streamline. They found good agreement with experiments in two-dimensional (partly) flexible indented channels [27].

The interesting dynamic phenomena of collapsible tubes are discussed in [28-31]. When the tube wall is partially collapsed strong oscillations may occur, even under steady flow conditions. The non-linear coupling between the fluid pressure and tube wall deformation can produce conditions in which high-grade stenoses may collapse [32]. We note that in the late systole the compression of the artery in a myocardial bridge may cause conditions where the vessel is entirely closed and where the flow limiting effect during re-opening becomes significant.

### Clinical situation

Under normal circumstances, coronary arteries have diameters large enough to transport sufficient amounts of oxygen to myocardial cells. Increases in myocardial oxygen demand, e.g. during exercise, are met by increases in coronary artery blood flow because – unlike in many other organs – extraction of oxygen from blood cannot be increased. This is in part mediated by increases in diameters of small intra-myocardial arteries. The large proximal (epicardial) coronary arteries contribute only a small fraction of total vascular resistance and show little variation in diameter during the cardiac cycle in any given metabolic steady state. Under maximum arteriolar vasodilation, the resistance imposed by the myocardial bed is minimal and blood flow is proportional to the driving pressure.

**Figure 1:**
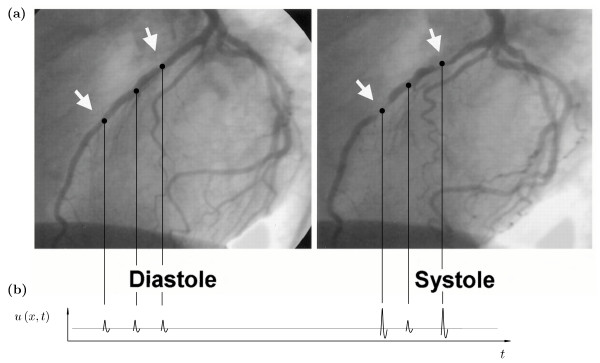
**Coronary angiogram of two myocardial bridges in the LAD**. (**a**) Coronary angiogram of two myocardial bridges in the left anterior descending (LAD) branch (arrows) in diastole (left) and systole (right). Compression of the artery during the hearts contraction phase, i.e. systole, is a characteristic finding in myocardial bridging (see text and [6] for details), (**b**) Diastolic lumen dimensions and flow velocity are normal, while systolic flow velocities are increased within the bridged segments.

The most common cause of an impaired ability to match oxygen supply and demand is coronary atherosclerosis, a disease that eventually leads to fixed coronary artery lumen narrowing, impaired coronary blood flow and potentially myocardial infarction. However, some people present with chest pain caused by phasic lumen obstruction due to myocardial bridging, first mentioned by *Reyman *in 1737 [33]. In this anatomic variant, a coronary artery segment courses underneath myocardial fibres resulting in vessel compression during systole, i.e. the myocardial contraction phase [6]. Myocardial bridges are most commonly found in the mid LAD, 1 *mm *to 10 *mm *below the surface of the myocardium with typical length of 10 *mm *to 30 *mm*. An angiogram of two myocardial bridges in series shown in Figure 1(a).

Although coronary blood flow occurs predominantly during diastole, i.e. the filling phase of the hearts chambers, total blood flow may nonetheless be reduced partly because vascular relaxation may extend significantly into diastole, the myocardial relaxation phase. Within the bridged segments permanent diameter reductions of 22 – 58% were found during diastole, while in systole the diameters were reduced by 70 – 95% [5]. A schematic drawing of the increased flow velocities (*cm/s*) during systole (31.5 within versus 17.3 proximal and 15.2 distal) is given in Figure 1(b).

From a medical point of view coronary angiography is limited in its ability to determine the physiologic significance of coronary stenosis [34,35]. As a result, intracoronary physiologic measurement of myocardial fractional flow reserve was introduced and has proven to be a reliable method for determining the functional severity of coronary stenosis. Previous studies have shown that the cut-off value of 0.75 reliably detects ischaemia-producing lesions for patients with moderate epicardial coronary stenosis [36]. The assessment of the FFR is independent of changes in systemic blood pressure, heart rate, or myocardial contractility and is highly reproducible [37]. The concept of coronary pressure-derived FFR has been extensively studied [13,38-40], clinically validated [41] and was found to be very useful in identifying patients with multi-vessel disease [42], who might benefit from catheter-based treatment instead of surgical revascularisation. As in [38], we have defined the pressure derived FFR as the ratio between the pressures measured distal to and proximal to the myocardial bridge during maximal hyperaemia. The exact locations of pressure measurement are given later in the text.

In summary myocardial bridges are characterised by a phasic systolic vessel compression with a persistent diastolic diameter reduction, increased blood flow velocities, retrograde flow, and a reduced flow reserve [5]. The underlying mechanisms are fourfold. Firstly the discontinuity causes wave reflections, secondly the dynamic reduction of the vessel diameter produces secondary flow, thirdly there is evidence for flow separation in post stenotic regions [43,44] and finally at severe deformations (or elevated flow velocities) the artery may temporally collapse [32].

### Objective

To ascertain the severity of the disease it is often desirable to have simple models to predict the pressure drop characteristics. A review of the available literature reveals that a few models exist, which are able to predict pressure drop (or friction factor) in non-circular ducts [45] (and references therein). However, the available models are for fully developed flow in non-circular ducts with fixed walls and mostly require tabulated coefficients.

In this study we intend to investigate physiological relevant cases of developing blood flow through a myocardial bridge located in the middle segment of the LAD. A prior model with similar geometry [46], but based on the assumption of fully developed Hagen-Poiseuille flow, was used to determine the influence of severity, length and degree of deformation and vascular termination on the flow. The results, however, indicated that the pressure drop was not realistic, which we assume is mainly due to negligence of entrance and separation losses. The system studied herein is the developing flow of an incompressible, viscous fluid through a network of elastic tubes in response to the aortic pressure. The tube characteristics and fluid properties are known, the developing flow conditions, the pressure response and mean FFR are desired quantities. We primarily substantiate the influence of frictional losses and separation losses on the translesional pressure drop and we calculate the mean fractional flow reserve to determine the haemodynamic relevance of the myocardial bridge. Further, we examine the consequence of external deformation on the wall shear stress distribution along the vessel.

## Methods

The fluid mechanics involved in flow through a myocardial bridge is complex, because of the three dimensionality of the deformations, coupling of the fluid with the arterial wall and flow separation. To understand the complicated behaviour of the tube flow, it is convenient to start with a one-dimensional approximation, which qualitatively predicts the overall aspects in spatially averaged flow variables. It is commonly derived by using equations for mass and momentum conservation and can be found in several places [47-50]. Because the equations are in common use, we will shortly repeat the main assumptions made. However, we will point out the differences introduced by the wall deformation, the entrance type of flow and flow separation. Firstly, we assume that blood flow in reasonably large vessels can be modelled as incompressible, Newtonian fluid with constant density *ρ*_0 _= 1055 *kg/m*^3 ^and constant dynamic viscosity *μ *= 4 *mPa s*; the kinematic viscosity is defined as *ν *= *μ/ρ*_0_. The Reynolds number *Re*_*d *_= *ud/ν*, based upon the vessel diameter, *d *and the mean flow velocity *u*, is below 2000 in non-diseased locations of the coronary arteries, so that the flow can be assumed to be laminar [9].

### The basic geometry

**Figure 2:**
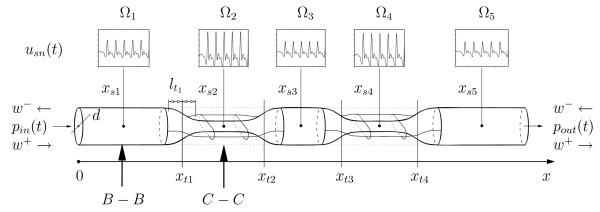
**Simplified geometry of the myocardial bridge**. Schematic anatomy of a double myocardial bridge. The control segments Ω_*n *_are equally spaced. Observation locations for haemodynamic properties are in the centre of each segment at *x*_*sn*_, transitions between the segments are at *x*_*tn*_. The transitions length between the segments is denoted by *t*_*ln*_. The graphs above each segment illustrate the mean flow velocity *u*_*sn*_. To illustrate the deformation we have indicated two cross-sections *B *- *B *and *C *- *C *in the circular and non-circular segments respectively (see Figure 3).

Theories of longitudinal waves in tubes, with or without non-uniformities, non-linearity and frictional dissipation, are based on the idea that variation of the pressure in an axial cross-section is negligible. If the internal and external pressure along the main flow axis, *x*, of the artery at time *t *are given by *p*_*int *_(*x*, *t*) and *p*_*ext *_(*x*, *t*) respectively, the transmural pressure across the wall of a tube is *p*_*tm *_= *p*_*int *_- *p*_*ext*_. Henceforth we assume that the external pressure is constant in space and time and consequently it is the internal pressure whose gradients produce fluid acceleration. Our first geometrical simplification for modelling blood flow in arteries is that the curvature along the axis of the tube is assumed to be small everywhere and that the flow in the cardiovascular system is unidirectional, so that the problem can be defined in one space dimension along the *x*-axis. According to this we have simplified the anatomy of the myocardial bridge as shown in Figure 2.

**Figure 3:**
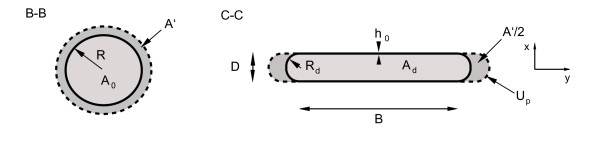
**Deformation cross-section**. This figure illustrates the deformation geometry of a circular, linear elastic tube neglecting the bending stress inside the wall. Cross-section *B *- *B *(left) shows circular expansion under pressure and the cross-section *C *- *C *illustrates the geometry under external deformation (right). The equilibrium cross-sectional area *A*_*d *_is shaded in light grey, while the perturbation area *A' *is shaded in dark grey. The dashed lines indicate expansion under pressure.

The two arrows in Figure 2 denote the location of either circular (*B *- *B*) or oval (*C *- *C*) cross-section of the tube. Due to the fact that the wall thickness, *h*_0_, is small compared to the bending radius *R*_*d *_(*h*_0_*/R*_*d *_≪ 1), we assume that the bending stress inside the wall is negligible. Consequently the cross-section of a circular tube (Figure 3 (left)) under deformation in *z*-direction, is given by the composition of a rectangle with two semicircular ends as illustrated in Figure 3 (right). This is consistent with the predominately eccentric deformation of bridged segments found in [3]. We note that negligence of bending stress causes the tube to collapse significantly earlier, i.e. the assumption is only satisfied if *p*_*tm *_≥ 0.

The deformation distance between the squeezing muscles and the breadth of the flat portion are denoted by *D *(*x*) and *B *(*x*, *t*) respectively. The equilibrium geometry of the cylindrical tube in Figure 3 (left) is characterised by the inner radius, *R*_0_, the circumference *U*_0 _= 2 *π R*_0 _and the cross-section *A*_0 _= *π * R_0_^2^.

However the equilibrium cross-sectional area of the deformed tube is *A*_*d*_(*x*, *t*) (see Figure 3 (right)). The total cross-sectional area in the *yz*-plane of the tube is defined by *A *(*x*, *t*) = ∫_*A *_*da *and the actual circumference is *U*_*p*_(*x*, *t*) = 2(*π R*_*d *_+ *B*). Consequently the average flow velocity *u *(*x*, *t*) = 1/A ∫_*A*_*ν*_*x*_*da*, where *ν*_*x *_is the local value of the flow velocity in axial direction. The volume flux across a given section therefore is *q *(*x*, *t*) = *A u*.

As shown in the angiography 1 and Figure 2 the coronary arteries in myocardial bridges are structured by several wall deformations. Their number, degree and extension may independently vary with time, so that the axial curvature of the arterial wall for each of the *n *= 1...*N *myocardial bridges in series is characterised by *N *functions. The deformation is specified by a parameter *ζ*, defined as *ζ *= *R*_*d*_*/R*_0_, which is chosen in the stenosis n to vary with time as



where *g*_*n*_(*t*) are periodic functions describing the temporal contraction of the muscle fibres. *ζ*_*systole *_and *ζ*_*diastole *_are fixed geometrical parameters between 0 and 1, specifying the degree of systolic and diastolic deformation respectively. We note that in the centre of the deformation *ζ *(*x *= *x*_*s*2_) = *ζ*_0 _= *R*_*d*_*/R*_0_, i.e. the degree of deformation increases with decreasing *ζ*_0 _and consequently *ζ*_*systole *_<*ζ*_*diastole*_. To represent the time dependence of the deformation found in intra-vascular ultra-sound (IVUS) measurements [51], a synthetic deformation waveform *g*_*n*_(*t*) given by m = 1..3 sine/cosine harmonics is used.



**Figure 4:**
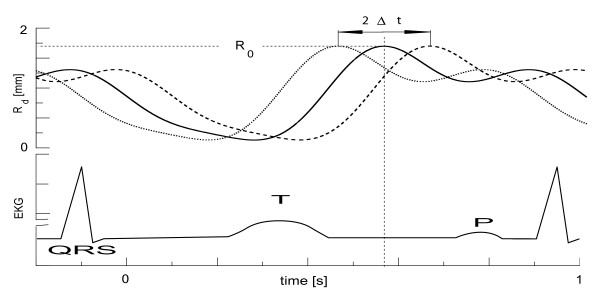
**Deformation function**. The variation of the bending radius *R*_*d*_(*x*_*S*2_, *t*) in the centre of the deformation is plotted with respect to the cardiac cycle, represented by a EKG trace. The time shift with respect to the cardiac cycle is denoted by Δ*t*, i.e. the solid line has zero time shift, while the dotted and dashed lines are shifted by ± 100 *ms *respectively. The periodic function *g*(*t*) in equation 3, was chosen according to IVUS measurements in [51].



Here Δ*t*_*n *_is the time shift for each deformation with respect to the cardiac cycle (see Figure 4) and *φ*_*m *_are the phases in radian, chosen to be *φ*_1 _= 3.5, *φ*_2 _= 1.5, and *φ*_3 _= 3.9. The axial curvature of each deformation is approximated by two hyperbolic tangent functions, so that

where *x *is the axial coordinate and *x*_*tn *_are the transition locations. Equation (3) smoothes the transition between the segmental domains Ω_*n *_by a transition length 1_tn_. The actual state of deformation can also be expressed by the ratio, *ε*, based upon the half-axes of the non-circular duct.



### The pressure-area relationship

Due to its complicated structure, it is difficult to provide a synthetic mathematical description for the mechanical behaviour of vessel walls. Here, we focus on the most relevant structural features and the simplest mathematical model for arterial tissues. In the following we derive an algebraic pressure-area relationship for a vessel under external deformation. In this context the distensibility characterises the relative change in cross-sectional area with respect to the pressure increment for a given deformation according to *A *= *A *(*ζ*, *p*). If we assume that *A' *is the perturbation about the equilibrium area *A*_*d*_, the total cross-sectional area can be written as *A *(*ζ*, *p*) = *A' *(*ζ*, *p*) + *A*_*d *_(*ζ*). For a homogeneous, thin-walled (*h*_0_*/R*_0 _≪ 1), linear elastic tube, the stresses in circumferential direction are large compared to stresses in longitudinal direction and the hoop stress *per unit length *of the tube is



where *E *is the elastic modulus and *σ *is the Poisson ratio, which for practically incompressible biological tissue is approximately 0.5. The circumferential strain in the vessel wall is



Equation (5) can be rearranged into the form



whereas Equation (6) leads to an expression for the pressure dependence of the breadth, *B*, of the flat portion of the tube.



The total cross-sectional area is

*A *(*R*_*d*_, *p*) = *π *R^2^_d_ + 2 *B *(*R*_*d*_, *p*) *R*_*d*_.     (9)

In the unperturbed state, the cross-section is

*A*_*d *_(*R*_*d*_) = *R*_*d *_*U*_0 _- *π *R^2^_d_.     (10)

We can finally write the pressure induced perturbation as



It should be noted that under the assumption of linear elastic material with constant elastic modulus, equation (9) and (11) have the property that the area increases linearly with transmural pressure. Real arteries, however, resist over-expansion by having an incremental elastic modulus, *E*(*ε*), that increases with increasing strain [52]. It should be further noted that the area perturbation in equation (11) is not only dependent on pressure variation but also on the degree of deformation through *R*_*d*_. By using equation (7) and (9) we can finally write the pressure-area relation



### Elastic properties of the coronary arteries

The elastic properties for a given section of the circular tube are obtained by using estimates for the volume compliance as suggested in [53], where the empirical approximation in exponential form is



In these estimates *k*_1_, *k*_2_, and *k*_3 _are constants. With data for the volume compliance from *Westerhof *[54], *Stergiopulos *[55] and *Segers *[56] we obtain *k*_1 _= 2.0 * 10^6 ^Kg s^-2^m^-1^, *k*_2 _= -2.253 * 10^3 ^m^-1^, and *k*_3 _= 8.65 * 10^4 ^Kg s^-2^m^-1^. A functional relationship for the wall thickness subject to the vessel radius can be found in [57], where

*h*_0 _= *a *R_0_^b^.     (14)

The parameters for *a *= 3.87 and *b *= 0.63 were obtained by a logarithmic fit to data including vessel radii between 100 *μm *and 3000 *μm*. Equations (13) and (14) are used to determine the wall thickness and elastic properties of the vessel, if the radius is known. The assumption of small bending resistance is well satisfied if *a *R_0_^(b-1)^ ≪ 1, which for typical vessels under consideration is below 0.21.

### Interaction of viscous boundary layer and inviscid core flow

In the following we investigate the solutions of the unsteady boundary layer equations by using an approximate integral method proposed by Veldman [58]. For this purpose, the potential flow of the two-dimensional equations governing the unsteady incompressible laminar boundary layer flow in axial symmetry is assumed to be in power-law form. By the introduction of similarity variables and the assumption that the evolution of the velocity profile is weakly dependent on *x*, the boundary layer equations reduce to the Falkner-Skan equation. Based on this ordinary differential equation, closed form solutions to the von Kármán integral momentum equation are obtained by a curve fit representation. The steady skin friction coefficients and the non-linear momentum correction coefficients corresponding to the velocity distributions are obtained and compared with known results. In particular the results of the steady solution are found to compare favourably with the Blasius solution and values for fully developed flow.

#### Boundary layer equations

The notion of the boundary-layer approximations was first developed by Ludwig Prandtl in the early 1900's. These well-known approximations [59] are applied widely in fluid mechanics. As the flow rate in the tube increases (i.e. high Reynolds number) the boundary-layer approximations become increasingly valid. The derivation of the axial boundary layer equations was first given by Mangler (1945) and can be found in [60]. In cylindrical coordinates (*x*, *r*, *φ*) with the corresponding velocity components (*ν*_*x*_, *ν*_*r*_, *ν*_*φ*_) and circumferential velocity *ν*_*φ *_= 0 (no swirl in S), they are







with     *ν*_*x*_(*x*, *R*_*d*_, *t*) = 0     *ν*_*r*_(*x*, *R*_*d*_, *t*) = *ν*_*w*_(*x*, *t*),     (18)

where *R*_*d*_(*x*, *t*) is the body shape along a *xr*-section of the tube or local surface radius measured from the axis and *τ *represents the shear stress, which is defined as *τ *= *μ* ∂V_X_/∂r. The derivation assumes that *R*_*d *_is much larger than the boundary layer thickness *δ*. The three-dimensionality of deformation generally makes it difficult to find a satisfactory solution for every compartment of the neither circular nor flat duct. However, considering severe deformation (e.g. *ζ*_0 _= 0.2) the circumferential length of the flat portion of the vessel exceeds the circumferential length of the circular portion by a factor of four (), thus we assume plane wedge flow for the calculation of viscous forces. Consequently *ν*_*w *_is taken to be the velocity component normal to the flat portion of the wall, which is *ν*_*w *_= -∂Rd/∂t. At the edge of the boundary layer, the free-stream velocity *V *(*x*, *t*) must be related to the pressure by the potential flow relation



#### Integral momentum equation

The integral momentum relation of von Kármán (1921) is obtained by multiplying the continuity equation (15) by *u *- *V *and subtracting it from the momentum equation (16). Integration over the bending radius and introduction of the integral relations for the displacement thickness



the momentum thickness



and the total displacement thickness δ** = δ* + *θ*



leads to



with     *u*(0, *t*) = *V*(0, *t*),     *θ*(0, *t*) = δ*(0, *t*) = 0,     (24)

where *c*_*f *_is a non-dimensional friction factor defined as



The only difference to plane flow is the term involving *∂R*_*d*_*/∂*_*x*_. If *R*_*d *_→ ∞ or *∂R*_*d*_*/∂x *→ 0, equation (23) reduces to the von Kármán integral momentum equation for plane flow. Compared to the frictional term c_f_/2 the influence of the term involving ∂*R*_*d*_*/∂x *on the boundary layer properties is indeed small (below 0.3%), thus disregarding the term for simplicity is appropriate. The boundary conditions in (24) assume a uniform inflow profile.

#### Falkner-Skan equation

Suitable solutions to the boundary layer equations in either plane or axial symmetry are found by the introduction of the stream function. A suitable coordinate transformation turns the equation for the stream function into the Görtler equation (derivation see [59]). The following approach mainly consists in assuming that the flow is locally self-similar and that it depends weekly on the coordinate *x*, so that the velocity profiles can be mapped onto each other by suitable scaling factors in *y*. Falkner and Skan have found a family of similarity solutions, where the free-stream velocity is of the power-law form

*V*(*x*) = *Cx*^*n*^,     (26)

with a constant *C *and the power-law parameter *n*. The similarity variable *η *~ *y/δ*(*x*) is set as



where *f*(*η*) is the dimensionless stream function and the prime refers to derivative with respect to *η*. The coordinate normal to the plate is denoted by *y*. However there are other general similarity solutions including the temporal dependence of the profile evolution [61]. The above similarity variables turn the boundary layer equations into a non-linear ordinary differential equation of order three, which is known as the Falkner-Skan-Equation





The parameter *β *is a measure of the pressure gradient *∂p/∂x*. If *β *is positive, the pressure gradient is negative or favourable, *β *= 0 indicates no pressure gradient (i.e. the Blasius solution to flat plate flow) and negative *β *denotes a positive or unfavourable pressure gradient. We note that by assumption, *β *should vary slowly with coordinate *x*. The solutions are found numerically by a shooting method with *f''*(0) as free parameter by Hartree [62]. To avoid extensive calculations we follow a curve fit representation of three quantities extracted from solutions of the Falkner-Skan equation used in [19]:







The relation to flat plate flow is generally given by the shape factor, which is defined as *H *= δ^*^/0, whereas *H*_0 _is the equivalent value for plane flow over a flat plate. The curve fits provide a good approximation for values of H between 1 and 20. At the separation point the wall shear stress vanishes, i.e. ∂V_X_/∂r = 0, which is equivalent to a shape factor *H *= 4. Relation (32) is not required for the calculations, however it is useful to predict the actual boundary layer thickness *δ*_99_, where the fluid velocity differs by 1% from the free stream value. The relation for the shear stress given in [19] is



consequently the friction factor is



**Figure 5:**
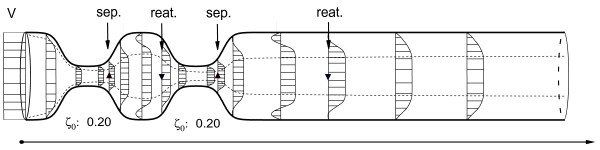
**Boundary layer evolution in the myocardial bridge**. Illustration of boundary layer separation in a series of two myocardial bridges at a deformation of *ζ*_0 _= 0.2; geometry and boundary layer thickness are displayed in realistic proportions, the velocity profiles are schematically drawn. The inflow profile is uniform with velocity *V*. We note that the extension of the separation zones differ, because the second myocardial bridge experiences different flow conditions.

A schematic illustration of the actual flow profile along the tube axis is shown in Figure 5. We have chosen a uniform inflow profile with velocity *V*(0, *t*). The boundary layer (dashed line) grows from the leading edge, decreases in the converging part, while it grows in the divergent part of the tube. The upward triangles (▲) denote the point of separation, while downward triangles (▼) indicate the reattachment of the boundary layer. After separation the flow field can be seen as a top hat profile in the centre and a recirculation zone close to the walls. Due to the adjacent converging part the reattachment is forced early, because fluid is accelerated. In contrast the reattachment after the second diverging part takes place further downstream.

#### Averaged flow equations

The simultaneous viscid-inviscid boundary layer approach assumes an inviscid core flow, which follows equation (19) and a viscous boundary layer, which may be found by the solution of equation (23). The one-dimensional equations commonly used to simulate unsteady, incompressible blood flow in elastic tubes with frictional losses [53,63] are given in averaged flow variables as





where *F*_*ν *_is the viscous friction term and *χ *is the momentum correction coefficient. The viscous friction term is defined as



and the momentum correction coefficient is



We rearrange the equations written in area and flow rate in terms of area and area-averaged axial flow velocity so that





The derivative of *A*_*d *_with respect to time in equation (39) is a prescribed function depending on *R*_*d*_(*x*, *t*). It is responsible for the volume displacement caused by the forced deformation of the tube.

#### Hagen-Poiseuille viscous friction and momentum correction

The determination of viscous friction factor and momentum correction coefficient requires knowledge about the velocity profile. For pulsatile laminar flow in small axially symmetric vessels a flow profile of the form



is used [50]. Here *û *is the free stream value of the axial velocity and *R *is the actual radius of tube, while *γ *is the profile exponent, which for a Hagen-Poiseuille flow profile is equal to two. Consequently the friction term is given by

*F*_*ν *_= -2 *π ν*(*γ *+ 2) *u *= *K*_*ν *_*u*,     (42)

whereas the friction coefficient for a parabolic profile is *K*_*ν *_= -8 *π ν*. The corresponding momentum correction coefficient is given by



which is 4/3 in the parabolic case. Such factors can be used for the purpose of correlating other variables as well as for direct calculation of pressure drop. We note that in the presence of a stenosis the total losses under the assumption of Hagen-Poiseuille flow are underestimated [55]. This comes mainly from underestimating the viscous forces and disregarding the losses caused by flow separation at the diverging end of the stenosis [64,65].

#### Boundary layer derived viscous friction and momentum correction

Developing flow conditions in ducts of multiply connected cross-sections generally make it difficult to use the right friction factor. A variety of cross-sections are discussed in [45]. In those situations the similarity parameters are preferably based on the free stream velocity and the square root of the cross-sectional flow area as characteristic length scale i.e. . Consequently we define the Reynolds number , the Womersley number or frequency parameter  and the Strouhal number . Here *ω *is the angular frequency defined as *ω *= 2*πf*, with *f *the base frequency of pulse wave oscillation. In other words we have multiplied *Re *and *Sr *by a factor of , while *Wo *is multiplied by . In the calculations we have given the Reynolds number inside the stenosis, *Re*_*st*_, based on .

**Figure 6:**
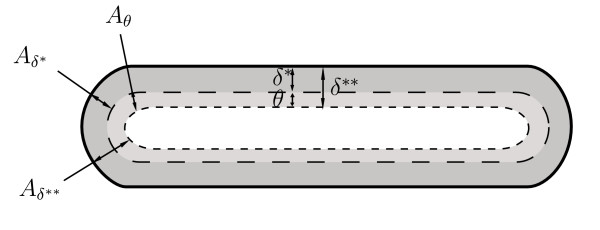
**Displacement thickness and displacement area in a deformed tube**. Illustration of the displacement thickness δ*, the momentum thickness *θ *and the total displacement thickness δ** in a deformed cross-section. The related areas are the displacement area *A*_δ* _(dark grey), the momentum area *A*_*θ *_(light grey) and the total displacement area *A*_δ** _= *A*_δ* _+ *A*_*θ *_(light + dark grey) respectively.

As previously mentioned the surface line of the flat portion of the non-circular duct dominates the circular portion at severe deformations, so that the computation of viscous forces is based on plane wedge flow. Consequently the thickness of the boundary layer is estimated in the *xz*-plane. Further we assume that the boundary layer has constant thickness along the circumference as illustrated in Figure 6. The latter assumption allows a simple derivation of the momentum correction coefficient and the viscous friction term. Integration over the cross-section leads to geometric relations for the areas occupied by the displacement thickness δ* and the total displacement thickness δ** in that cross-section. They are expressed as

*A*_δ* _= 2B δ* + *π *[R^2^_d_ - (*R*_*d *_- δ*)^2^],     (44)

*A*_δ** _= 2B δ** + *π *[R^2^_d_ - (*R*_*d *_- δ**)^2^],     (45)

It is obvious that *A*_*d *_> *A*_δ** _and *A*_*d *_> *A*_δ* _have to be satisfied to make sure that the flow is not fully developed. The momentum correction coefficient can be found by satisfying mass conservation for the mean flow and the core flow by

*V *(*A *- *A*_δ*_) = *A u*,     (46)

which can be used together with equation (22) and (45) in the definition for the momentum correction (43), so that



The uniform inflow profile is identical to *χ *= 1, while the developing profile reaches its far downstream value of 1.39 after the entrance length within less than 4.5% from the analytical solution for the parabolic flow profile given in equation (43). We note that in the linearised system the total cross-section *A *in equation (47) is replaced by *A*_*d*_. According to equation (34) the friction factor built with the pressure dependent surface line, *U*_*p*_(*x*, *t*) is



Computations in a uniform tube show good agreement to the friction factor of the parabolic profile given in equation (42). After the entrance length the friction factor computed via the boundary layer theory reached its far downstream value to within 7%. Additionally the Fanning friction factor Reynolds number product in a deformed vessel geometry agrees well with experiments carried out in [45]. In contrast to other proposed models the underlying model does not require knowledge about the fully developed friction factor Reynolds product *c*_*f *_*Re*_*fd *_[45], or the incremental pressure drop factor *K*_∞ _[66,67]. The above results for momentum correction and viscous friction quantify the entrance conditions typically encountered in studies of the arterial system.

#### Validity

The stationary boundary layer approximation becomes increasingly valid if the Reynolds number increases and when the ratio of unsteady forces to viscous forces given by the Womersley number is small. In the left coronary artery the values for Womersley and Strouhal number, built with pulsatile frequency were around 4 and below 0.2 respectively. The approximation of the actual flow profiles by near equilibrium flow profiles is justified for stationary flow, however, for time dependent flow the period *T *of the deformation function in equation (3) has with be large compared to the viscous diffusion time through the boundary layer: *t*_*d *_= δ^2^/V ≪ *T*. This is well satisfied for the situation under consideration, where *t*_*d *_in the centre of the deformation was between 4.4 * 10^-3 ^*s *and 0.2 *s *at values of *ζ*_0 _of 0.25 and 1 respectively.

### Computational domain

**Figure 7:**
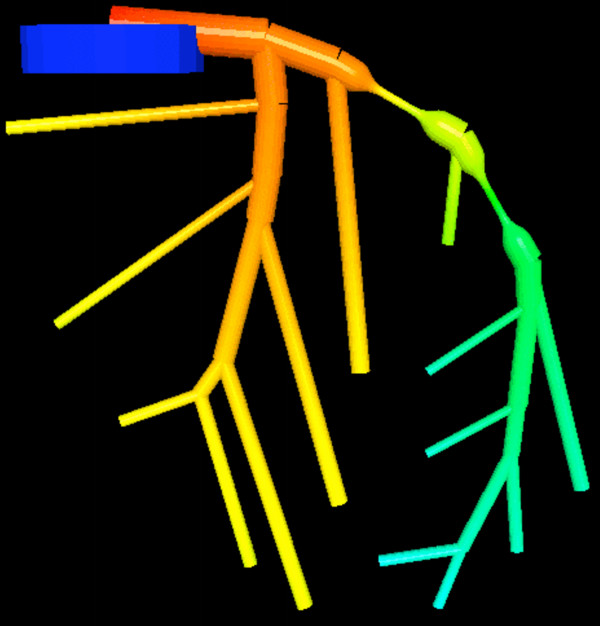
**Computational domain**. Structure of the 27 main segments of the left coronary arteries. The dimensions of a normal anatomic distribution of coronary artery segments are based on 83 angiographies by *Dodge *et al. [68, 69]. A series of two consecutive myocardial bridges is situated in the mid LAD. In this case we have coloured the segments according to the pressure distribution calculated by the proposed boundary layer model (high pressure is coloured red, while low pressure is coloured blue). The dark blue, vertical cylinder indicates a segment of the ascending aorta.

Based on 83 angiographies, *Dodge *et al. [68,69] presented a normal anatomic distribution of coronary artery segments and proposed a terminology, which we used for our model of the left coronary artery (LCA): the left main coronary artery (LMCA) bifurcates into the left anterior descending artery (LAD) and the left circumflex artery (LCxA). The main branches of the LAD include the 1^*st*^, 2^*nd *^and 3^*rd *^diagonal branch (Dl, D2, D3) and the 1^*st*^, 2^*nd *^and 3^*rd *^septal branch (S1, S2, S3). The main branches of the LCxA include the 1^*st *^and 2^*nd *^obtuse marginal branches (OM1, OM2). The exact intrathoratic location and course of each one of the 27 arterial segments and branches of the LCA are illustrated in Figure 7. We note that in the present 1D approximation the arterial tree is composed of tubular entities. The branching angles in figure 7 serve only artistic purposes.

### Boundary conditions

**Figure 8:**
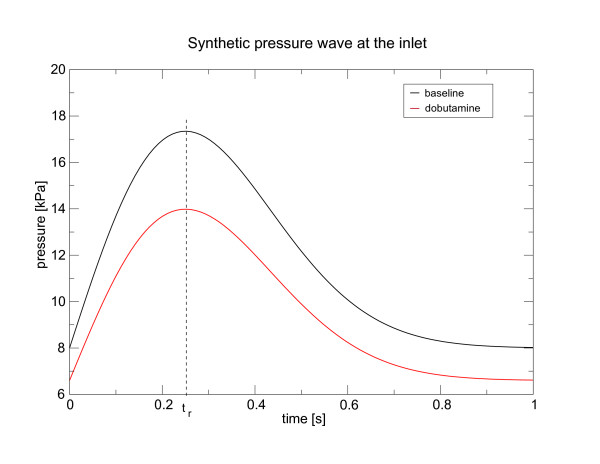
**Synthetic pressure wave at the inlet**. Synthetic pressure wave at the inlet to the left coronary tree are modelled by equation (49). We have chosen the parameters according to measurements in [38]. The baseline (black line), is represented by a stationary pressure *p*_*s *_= 8 *kPa *and a pressure amplitude of *p*_0 _= 7 *kPa*, while for the inlet pressure under dobutamine (red line) *p*_*s *_= 6.6 *kPa *and *p*_0 _= 5.5 *kPa*. In both cases the raising time was *t*_*r *_= 0.25 *s*.

Because the coronary flow is primarily driven by the aortic pressure, the pulsatile inflow condition to the LMCA was represented by a periodic extension to a synthetic pressure wave in the exponential form



where *p*_*s *_is the static pressure and *p*_0 _is the amplitude of the exponential waveform, while *t*_*r *_is the rising time. We have chosen the parameters according to measurements in [38]. The baseline condition is represented by a stationary pressure *p*_*s *_= 8 *kPa *and a pressure amplitude of *p*_0 _= 7 *kPa*, while for the inlet pressure under dobutamine challenge we have chosen *p*_*s *_= 6.6 *kPa *and *p*_0 _= 5.5 *kPa*. In both cases the raising time was *t*_*r *_= 0.25 *s*. The pressure wave at the inlet is shown in Figure 8.

The branching conditions between the 1D entities are implemented by the requirement of constant pressure at the branching point and mass conservation throughout the bifurcation [63]. To avoid wave reflections at the ends of the tubes the boundary conditions are implemented by a characteristic system of one-way wave equations [46]. There are several ways to account for peripheral reflections at the terminals of a vascular network. In the current simulations we have implemented a three-element windkessel model for the termination of the left coronary arterial tree [70,71]. The main advantage of this model is to consider the compliant-capacitive effects due to micro-vessels and arterioles. To satisfy the Blasius solution at the leading edge of the tube, we have assumed a uniform flow profile at the entrance (V(0, t) = u(0, t)).

### Numerical implementation

Due to the non-linear terms in equation (23) and (40) the solutions for haemodynamically developing flows are generally more difficult to obtain than fully developed flows or oscillating flows with a frequency dependent Stokes boundary layer. Developing flows require simultaneous solution of the momentum equation (39), the continuity equation (40) and the integral momentum equation (23), together with the boundary conditions given in (49) and (24) respectively. The system of equations cannot be solved analytically, so that the interior domain was solved by a second order predictor-corrector MacCormack finite difference scheme with alternating direction for prediction and correction in each time step [72]. To implement the boundary and interface conditions it is convenient to disregard viscous friction and rewrite equation (39) and (40) in terms of characteristic variables [46]. The momentum correction factor in equation (47) and the viscous friction in equation (48) are given by the solution to the integral momentum equation (23) and the two curve fits to the Falkner-Skan equation in (30) and (31). They are solved by discretisation using the same second order MacCormack scheme and iterative solution of the resulting set of discrete non-linear equations by a combined root bracketing, interval bisection and inverse quadratic interpolation method of van Wijngaarden-Brent-Dekker. To start the computation the Blasius solution at each time step provides values for the boundary layer thickness a few grid points downstream of the entrance. The solution was applied to the steady integral momentum equation as boundary condition for δ*. Downstream marching the solution leads to the values of boundary properties along the tube axis, which through equation (47) and (48) provide the required values of momentum correction and viscous friction to the averaged flow equations respectively.

## Results

The derived model to simulate viscous friction and momentum correction in the entrance of a tube with varying cross-section was subsequently applied to a test geometry, which consists of a single elastic tube with either temporally fixed or dynamic indentations, and specific clinical situations in the LCA described in [38]. The simulations were carried out in temporal fixed and dynamic stenosis of different degree and extent. The Womersley and Strouhal number were around 4 and below 0.15 respectively, the Reynolds number in the stenosis varied from 850 to 1500. It was found that under application of a uniform flow profile at the entrance the core velocity in the vessel increases gradually downstream, indicating the development of a parabolic flow profile. For a Reynolds number of 2000 the distances over which the core velocity stabilised in the tube were very much shorter than at 800; fully in line with the dependence of the entrance length. However fully developed flow was not present in any of the cases.

The spatial resolution of the grid was adjusted to represent the curvature of deformation, where 4 gird points per mm was reasonable in any of the cases, the time resolution was chosen accordingly and ranged between 5 *μs *and 10 *μs*. These resolutions are sufficient to avoid numerical instabilities at severe deformations. The computational time required, for the simulation of one pulsatile cycle in the above described network of coronary arteries, was about 22 *min*. (Apple Power-Mac G5, 2 GHz).

In the following we address the flow limiting effect by the assessment of the pressure derived fractional flow reserve in different stenotic environments (test geometry and LCA). Further, we will discuss the pressure drop Δ*p*, the flow separation, the wall shear stress and the influence of external wall deformation. In some of the graphs we have shown the interval of possible solutions that may occur during the pulsatile cycle, i.e. the upper and lower envelope denoted by *Max*_*env *_(solid red line) and *Min*_*env *_(solid blue line) respectively.

### Modelling: test geometry

We have chosen the test geometry of the tube according to the size of the left main coronary artery with an internal diameter of 5 *mm*. To observe separation and reattachment we have adapted the length to the maximum extent of the recirculation zone, which was about 300 *mm *at 80% diameter deformation. At 20 *mm *downstream of the entrance, the diameter was abruptly decreased (*t*_*l *_= 4 *mm*) by 0 – 85%, with an extent of 30 *mm *(single stenosis), another constriction with the same length follows 30 *mm *further downstream (double stenosis). In addition the extent of the single stenosis (short) was varied by a factor of two (medium) and three (long), in the double stenosis we varied the separation distance between one and three dimensions of the stenosis. The wave velocity in those situations may take values as low as *c *= 5 *m/s *in uniform vessels, rising to values around *c *= 30 *m/s *in constricted vessels. Physiological peak flow velocities, V, are much smaller, generally around 0.5 – 1 *m/s*, but they can reach 2 – 3 *m/s *in parts of severe deformation.

**Figure 9:**
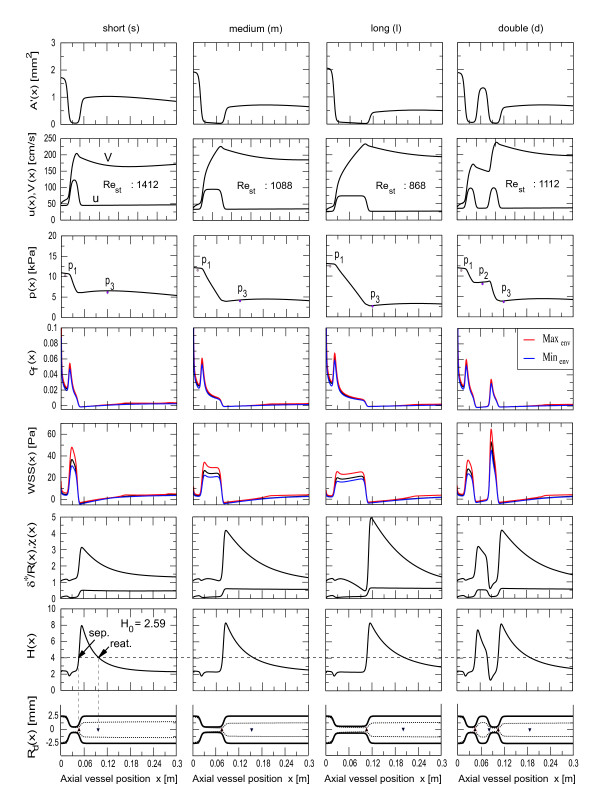
**Spatial dependence of flow variables in a tube with fixed deformation**. The area perturbation, flow velocity, pressure and friction coefficient, the wall shear stress, momentum correction factor, the normalised boundary layer thickness and the shape factor are plotted as a function of position in a vessel with fixed deformation of *ζ*_0 _= 0.2. Three stenoses of different extent (**s**), (**m**) and (**l**) and a series stenosis (**d**) are compared. The losses are about equal in the cases (**m**) and (**d**), which indicates that the pressure loss is mainly viscous. To indicate the flow characteristics in the stenosis we have used the Reynolds number *Re*_*st *_inside the deformation.

**Figure 10:**
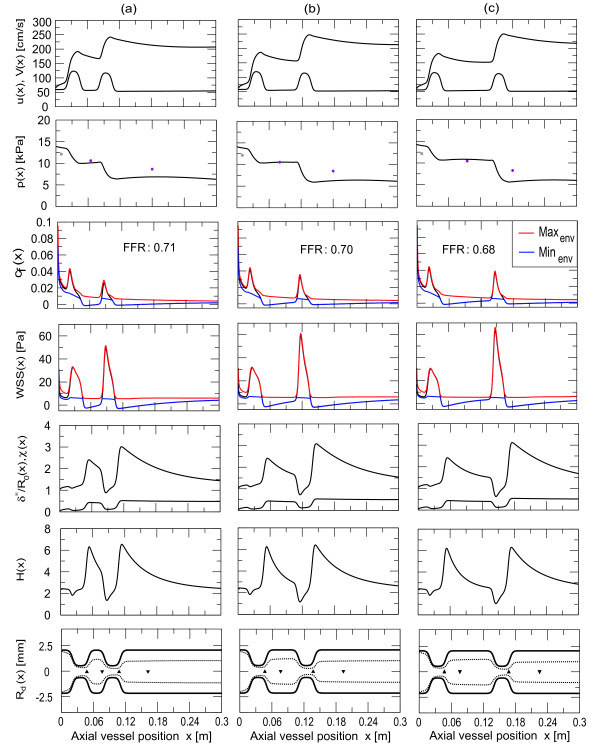
**Variation of stenosis distance**. In columns (**a**), (**b**) and (**c**) we compare a series of two dynamic stenoses, which are separated by one, two and three stenosis dimensions respectively. From the top we illustrate the flow velocity, the pressure, the friction coefficient, the wall shear stress, the momentum correction coefficient and normalised boundary layer thickness, the shape factor and the instantaneous geometry of the vessel. The boundary layer is illustrated as dashed lines. The upward triangles at the centreline of the vessel geometries indicate the separation point, while the downward triangles indicate the reattachment of the boundary layer. The FFR decreases with increasing distance between the stenoses.

The region around the diameter transition in the test geometries is shown in detail in Figure 9. The area perturbation *A'*, shown on the top of each column, indicates that the arterial deformation caused by pulsatile pressure is much smaller in regions where the tube is squeezed (about *A'/A *= 1 – 2%), while in circular segments *A'/A *= 8 – 9%. Due to incompressibility and mass conservation a sudden jump to a high velocity is seen at the narrowing. It appears that the stenosis influences the mean velocity only over a short distance upstream, while in the case of separation the core velocity slowly decays until reattachment, then it starts growing again until the flow is fully developed. The boundary layer separation in the post-stenotic region indicates an emerging top-hat velocity profile from the stenosis, with sideway counter-current flows. The decay in core flow velocity indicates shear widening of the top-hat until reattachment. Further downstream the core velocity is almost constant. It was found that the distance over which the outlet effect occurs is smaller for stenosis with small deformation and length and small Reynolds number. The core velocity influences the frictional losses and the extent of the separation zone in series stenoses, so that series stenoses cannot be represented by two similar building blocks. This becomes more evident through the fact that the entrance flow profile in the second stenosis changes, if the distance of the two stenoses is varied (Figure 10(a–c)). The downstream deformation is generally dominant in wall shear stress and frictional forces as well as in the extent of the post-stenotic separation zone. Further, it is likely that a downstream stenosis with equal deformation collapses significantly earlier, because the core flow velocity is increased, so that the transition from subcitical flow (*V *<*c*) to supercritical flow (*V *> *c*) happens earlier.

#### Pressure drop and flow limitation

Geometric influences on the pressure loss across series of stenoses have been studied in [15]. It was found that the pressure drop across severe stenoses is little affected by the eccentricity of the stenosis, dominantly affected by the severity and length of the stenosis, and affected by the Reynolds number only at low Reynolds number. The eccentricity of mild stenoses increases the likelihood of collapse of stenotic arteries, because the buckling pressure is reduced [28].

As in [1,8] we found that the pressure proximal to the deformation is increased if the degree of deformation or the length of deformation is increased. Compared to the mean reference pressure (no deformation) of 9.29 *kPa*, the mean pressure at the inlet for a deformation of *ζ*_0 _= 0.2 was 10.28 *kPa*, 11.53 *kPa *and 12.41 *kPa *in the cases (**s**), (**m**) and (**l**) respectively. Under these conditions the mean flow in the reference artery was 10.36 *cm*^3^*/s*. For fixed deformation of *ζ*_0 _= 0.2 we found 8.43 *cm*^3^/*s*, 6.38 *cm*^3^/*s*, 4.94 *cm*^3^/*s *and 6.66 *cm*^3^/*s *in the short, medium, long and double stenosis respectively. The pressure drop for the medium sized stenosis was at 7.52 *kPa *just below that of the double environment with 7.78 *kPa*, the pressure drop across the short stenosis was 4.15 *kPa *and across the long stenosis 9.82 *kPa*. In double stenoses the pressure drop across the proximal stenosis was 3.28 *kPa*, which is markedly less than 4.4 *kPa *at the distal stenosis. Complementary the values for the dynamic case are generally less pronounced, they can be found in Table 1.

**Table 1:** Mean flow quantities, pressure drop and fractional flow reserve for test geometry shown in Figure 9.

		fixed				dynamic				
measure	unit	short	medium	long	double	short	medium	long	double	ref

	[*kPa*]	10.42	11.63	12.48	11.46	9.55	9.84	10.07	9.80	9.29
Δ*p*_1–3_	[*kPa*]	4.15	7.52	9.82	7.78	1.89	2.74	3.47	2.87	1.23
*FFR*_*p*_	[*%*]	60	35	21	32	80	72	65	70	86
	[*cm*^3^*/s*]	8.43	6.38	4.94	6.66	9.90	9.40	9.01	9.46	10.36
*ū*_1_	[*m/s*]	0.43	0.33	0.25	0.34	0.50	0.48	0.46	0.48	0.53

However, the translesional pressure drop in a series of two stenoses with time dependent deformation in the entrance region of a tube shows further remarkable effects. The simulations indicate that in general the pressure drop cannot be obtained by a summation of pressure drops for single stenosis, since the proximal and distal stenosis influence each other unless the spacing between them exceeds some critical distance, which depends on the Reynolds number and deformation. Therefore several consecutive stenoses along the same epicardial artery require separate determination of stenosis severity. We found that when the two stenoses are close together, the pressure drop is approximately equal to that of a stenosis with twice the length of a single stenosis (see Table 1 and Figure 9 (**m**) and (**d**)). This can be explained by the fact that the friction coefficient in the recirculation zone between the two stenoses is small and consequently the pressure loss over that region is small. However, when the stenoses are separated by more than the length of the stenosis the flow is hardly affected by the second stenosis, because the core flow velocity and likewise the frictional force are increased (see Figure 10(a–c)), even though the entrance flow conditions are preserved. Coexistent is the increased reduction in shape factor and momentum correction coefficient, suggesting the non-linear influence in that region. The pressure drop over the downstream deformation is generally pronounced, however the distance between the deformations further increases the pressure drop and consequently the mean FFR is reduced. The values for the cases (**a**) to (**c**) are Δ*p*_*a *_= 3.45 *kPa*, Δ*p*_*b *_= 3.64 *kPa *and Δ*p*_*c *_= 3.89 *kPa *for the pressure drop over the downstream deformation and *FFR*_*a *_= 71%, *FFR*_*b *_= 70% and *FFR*_*c *_= 68% for the mean fractional flow reserve across the bridge. We note that these findings are based on the entrance type of flow, where the core flow velocity gradually changes and may not appear in fully developed flow, where the core flow velocity is a constant and identical to the maximum of Hagen-Poiseuille flow velocity.

#### Separation and reattachment

The core velocity in a uniform tube generally increases downstream of the entrance, however in presence of a stenosis the boundary layer thickness decreases at the inlet and rapidly increases in the diverging part of the constriction (see Figure 9). Separation occurs under the development of a top hat profile with sideway counter-current flow. At the separation point (▲) the boundary layer thickness increases and a sudden jump in *χ *is evident, because the integral of the actual velocity  over the area of the recirculation is close to zero and in contrast to the mean flow velocity the core velocity is increased. Therefore the non-linear term is pronounced in the separation region, while it is close to unity in converging regions. The momentum correction becomes markedly smaller than before the upstream stenosis. This indicates that the entrance profile into the second stenosis is almost flat, but has increased core velocity, while the counter current flow at the walls have disappeared. The reattachment of the boundary layer (▼) further downstream is caused by shear layer friction between the recirculation zone and the top-hat profile, which also causes the pressure to recover. Due to the increased momentum correction in that region the pressure in the non-linear case recovers more rapidly than in linearised computations, which causes earlier reattachment and consequently slightly smaller recirculation zones. The extent of the recirculation zone is primarily dependent on vessel deformation and Reynolds number, however, we found that the extent also correlates with the length of the constriction. Compared to the short deformation in Figure 9 (s) the tail of the shape factor curve drops below the critical value of 4 (condition for separation or reattachment) by a factor of about two and three later for the medium (**m**) and long (**l**) constriction respectively. In other words vessels with the same degree of stenosis, but with the stenosis having different curvatures and lengths, have recirculation regions that differ markedly in their extent. At deformations of 85% the recirculation zones had an extent of about 20 tube diameters in length. However the extent of the separation region was found to be strongly dependent on the degree of deformation and the Reynolds number. The separation point moves upstream, while the reattachment point moves downstream if the Reynolds number or deformation increases. A particularity of series stenoses is that the extent of the recirculation zone in the interconnecting segment is reduced. This is due to early reattachment caused by fluid acceleration in the converging part of the second stenosis. But nonetheless the core flow velocity is generally smaller compared to the downstream separation region.

#### Wall shear stress and friction coefficient

For steady flows the location of maximum wall shear is always upstream the neck of the stenosis (see Figure 9), and moves upstream as the Reynolds number increases. In series stenoses the WSS is significantly increased in the distal stenosis, while the friction coefficient is smaller there (see column (**d**)). Generally they have their maximum at the entrance of the stenosis and reduce towards the end of the stenosed section. Eventually they become negative after separation of the boundary layer. The increased boundary layer thickness in the downstream stenosis suggests lower retarding forces, however, the core flow velocity is increased there so that pressure losses are dominant there. Consequently the second stenosis can be seen as the more vulnerable, in wall shear stress and flow limitation. Likewise the mean flow velocity in a pressure driven vessel is dependent on the total after-load, the maximum values of wall shear stress are dominant in short constrictions (see column (**a**)), because the after-load is smaller and fluid velocity is increased compared to long constrictions (column (**l**)). Although the wall shear is increased in short constrictions, we observe that the peak of the viscous friction increases if the length of the constriction is increased. In flow driven vessels however the peak values are independent of the extent, because the flow velocity is equal in all cases (not shown here).

Wall shear stress oscillations have been observed for various downstream locations and severity of deformation. The amplitude of oscillation depends strongly on the axial position and the actual state of deformation. The wall shear stress is large in the entrance region of the deformation, fades towards the end and is negative in the separation region, so that the development of atherosclerosis is more likely in segments proximal to the deformation. Compared to wall shear stresses in non-diseased vessels (5 – 10 *N/m*^2^) vulnerable regions are endothelial cells in the throat of a strong deformation. They may experience wall shear stresses in excess of 60 *N/m*^2^. In series stenoses the stresses are largest in the downstream stenosis because the core flow velocity is increased there. Furthermore the wall shear stresses are no longer likely to be distributed evenly around the circumference of the vessel and may be particularly focused on the most vulnerable shoulder regions, marking the transition from normal to diseased artery wall. Further improvements for the prediction of the wall shear stress may be obtained by the introduction of a shear dependent model to predict the local blood viscosity.

#### Unsteady solutions

**Figure 11:**
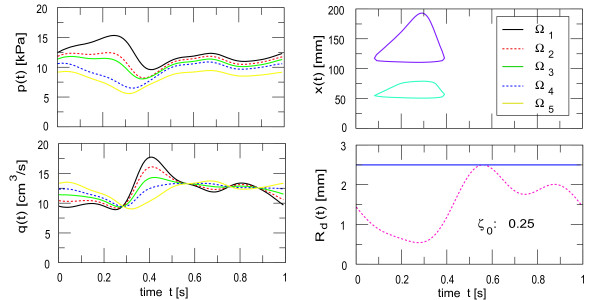
**Temporal evolution of flow variables**. The temporal dependence of pressure and volume flow (left) and the separation cycle during deformation (right) of a series of two myocardial bridges are illustrated. The dashed lines represent flow variables in segments with deformation (Ω_*s*2 _(red) and Ω_*s*5 _(blue)), while solid lines show the flow variables in segments without deformation (Ω_*s*1 _(black) proximal, Ω_*s*3 _(green) in the centre and Ω_*s*6 _(yellow) distal to the stenoses). Separation and reattachment during the cardiac cycle was observed for severe deformation between 0.1 *s *and 0.4 *s *(right).

Despite the assumption of strong coupling between the boundary layers and the core flow and the assumption of quasi-stationary evolution of the boundary layers, the time dependent motion of the wall under external deformation reproduces some remarkable characteristics of myocardial bridges. In Figure 11 we have shown the effect of temporal deformation onto the pressure and flow in the segments Ω_1–5 _of a series of two myocardial bridges (see Figure 2). The time dependence of the two separation zones, one between the two deformations and the other distal to the second deformation shows that separation occurs for deformations greater than about 40%. The separation cycle is present in the time interval of 0.1 *s *to 0.4 *s*. The maximum deformation during the cycle was 75% of *R*_0_, which was reached at 0.3 *s*. It is seen that during deformation the separation point moves somewhat upstream, while the reattachment point of the boundary layer moves farther downstream. The upstream separation zone (turquoise cycle) is spread over a region of 49.94 *mm *to 80.83 *mm*, while the downstream separation zone (purple cycle) is from 109.98 *mm *to 193.32 *mm*. The extensions of the two zones differ, because the upstream reattachment point is forced early due to accelerating fluid at the inlet of the downstream deformation and because the core velocity is generally larger further downstream. In each case however, a top-hat velocity profile develops in systole, producing large recirculation zones distal to the stenoses, which are delayed into diastole. The back-flow is developing earlier for severe deformation and is strongly dependent on deformation phase. For phase delayed deformation, say, by half the cycle time (here 500 *ms*), the flow patterns are noticeably different (not shown here). The top-hat profile is now present in diastole, the peak velocities and the viscous friction are less pronounced and the reverse flow region is smaller and mainly present in the diastole. The wall shear stress is dramatically reduced compared to the zero phase case, because deformation maximum falls together with the haemodynamic conditions present in diastole. Furthermore the mean FFR was increased to 0.65 compared to 0.6 of the zero phase case. This can be explained by the circumstance that no appreciable pressure drop was present at the downstream stenosis.

### Modelling: physiological basis

The simulation of clinical relevant cases requires a specific set of parameters, which however is not available in the literature. Due to this difficulty we compare the FFR range obtained with parameters for the length and degree of deformation given in [38]. The length of the myocardial bridge for the baseline and dobutamine case were 12 *mm *and 24 *mm *respectively. The values for the deformation were *ζ*_*baseline *_= 0.54 and *ζ*_*dobutamine *_= 0.32. To assess the dynamics, we have applied more physiological waveforms (aortic pressure conditions) to the inlet of the left main coronary artery (LMCA) (see Figure 8). The peak Reynolds and Strouhal numbers in the myocardial bridge were 815 and 1069, and 0.021 and 0.016 for the baseline and dobutamine case respectively. The Womersley number was 4.11.

#### Mean pressure drop

**Figure 12:**
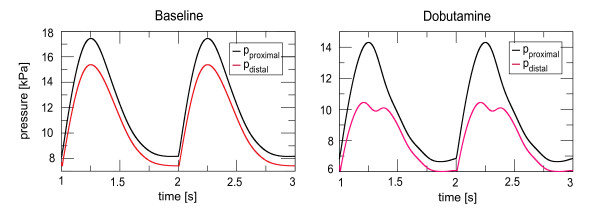
**Specific case of clinical relevance**. Intracoronary pressure at baseline and during dobutamine challenge. The proximal pressure, *p*_*proximal *_was taken from the inlet of the coronary tree (LMCA), the distal pressure, *p*_*distal *_in a segment 3 *cm *distal to the myocardial bridge. At baseline the mean FFR was 0.90, while during dobutamine challenge the mean FFR was 0.84. The corresponding pressure drops were Δ*p *= 1.25 *kPa *and Δ*p *= 1.54 *kPa *respectively.

The mean pressure drop was calculated by subtracting the average of a distal pressure wave, which was taken approximately 3 *cm *distal to the myocardial bridge, from the average inlet pressure at the LMCA. The proximal and distal pressure waves for the baseline and while dobutamine challenge are shown in Figure 12. It is seen that the baseline pressure is less affected, while the pressure under dobutamine challenge shows a pressure notch, which may appear if the deformation is dominant during systole (see earlier discussion on that in [46]). At baseline the mean pressure at the inlet was *p*_*p *_= 11.92 *kPa *and the mean distal pressure was *p*_*d *_= 10.67 *kPa*, so that the pressure drop was Δ*p *= 1.25 *kPa*, which compares well with the value of measurements mentioned above, where Δ*p *= 1.19 *kPa*. Under dobutamine challenge the corresponding values are *p*_*p *_= 9.64 *kPa*, *p*_*d *_= 8.1 *kPa *and Δ*p *= 1.54 *kPa*, which however are close to the measured values, where Δ*p *= 1.85 *kPa*.

We have further compared the translesional pressure drop across a series of two myocardial bridges resulting from Hagen-Poiseuille and boundary layer computations in three animations, one as reference, without a stenosis (Additional file 1), one assuming a Hagen-Poiseuille flow with a series stenosis, each of length 8 *mm *and deformation *ζ*_0 _= 0.25 (Additional file 2) and the same series stenosis with the boundary layer method described here (Additional file 3). It is clearly seen that the systolic pressure in Additional file 1 is uniformly distributed and fades towards the terminals of the network. In Additional file 2 we notice that during systole the pressure drops in both of the stenoses, so that the LAD branch is less distributed. However the boundary layer computations in Additional file 3 show that the pressure drop by the assumption of fully developed flow was underestimated in the case of developing flow conditions.

#### Fractional flow reserve

The flow limitation caused by epicardial stenoses is generally expressed by the flow based FFR, which is the ratio of hyperaemic myocardial blood flow in the presence of a stenosis to hyperaemic flow in the absence of a stenosis, *FFR*_*q *_= *q*_*s*_*/q*_*n*_, i.e. the flow based FFR is the fraction of hyperaemic flow that is preserved despite the presence of a stenosis in the epicardial coronary artery. However this definition is purely theoretic, because the flow without the stenosis is not known, so that for clinical purposes the ratio of hyperaemic flows with or without a single stenosis is derived from the mean distal coronary pressure *p*_*d *_to mean proximal pressure *p*_*p *_recorded simultaneously under conditions of maximum hyperaemia.

Neglecting correction terms the mean pressure-derived fractional flow reserve is *FFR*_*p *_= *p*_*d*_*/p*_*p*_. In the case of two consecutive stenoses however, the fluid dynamic interaction between the stenoses alters their relative severity and complicates determination of the FFR for each stenosis separately from a simple pressure ratio as in a single stenosis. Consequently the FFR determined for single stenosis is unreliable in predicting to what extent a proximal lesion will influence myocardial flow after complete relief of the distal stenosis, and vice versa.

Taking the pressure values resulting from boundary layer computations of the previous section, we obtain values for the mean pressure derived FFR of 0.90 and 0.84 for baseline conditions and under dobutamine challenge respectively. These values agree with the measurements in [38], where the values were 0.90 and 0.84 respectively.

**Figure 13:**
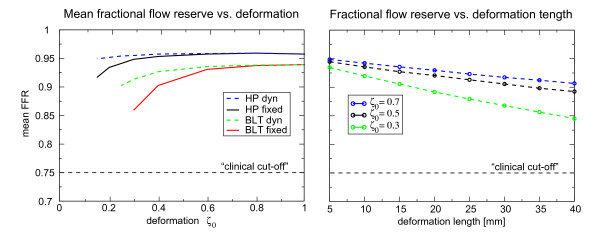
**Mean fractional flow reserve in dependence on degree of deformation (left) and segment length (right)**. The mean pressure derived fractional flow reserve of the baseline environment shown in Figure 8 is plotted as a function of deformation (left) defined in equation (3) and versus the segment length (right). The computations via the boundary layer theory (BLT) are compared to Hagen-Poiseuille (HP) flow profile (*γ *= 2). It is seen that the mean pressure derived fractional flow reserve is overestimated under the assumption of fully developed flow. Generally the mean FFR is larger in the dynamic case (dashed lines), because the pressure recovers during the relaxation phase. The segment length of three dynamic lesions at a deformation of *ζ*_0 _= 0.7, 0.5 and 0.3 was varied in a physiological range between 5 *mm *and 40 *mm*.

In Figure 13 we have shown the coronary pressure-derived fractional flow reserve for the baseline case as a measure of coronary stenosis severity (left) and in dependence on deformation length (right). In both plots we have used baseline inflow conditions with either fixed (solid lines) and dynamic walls (dashed lines). Initially we found that in developing flow conditions the FFR is overestimated by the assumption of Hagen-Poiseuille flow (HP), and, as expected, that the mean FFR in dynamic lesions is much larger than in fixed stenotic environment, because the distal pressure recovers during relaxation phase. The graph at the right shows that the mean FFR depends essentially linearly on deformation length for different severities, *ζ*_0 _= 0.7, 0.5, 0.3, suggesting that the losses are mainly viscous and that the non-linear term plays a minor role for observed deformations.

The values indicate that the FFR depends on the degree and length of the stenosis. As mentioned earlier the losses in fixed environments are more pronounced. Further, we point out that series stenoses separated by more than the length of the stenosis drop below the cut-off value of 0.75 markedly earlier than single stenosis with the same degree and extent.

#### Pressure-flow relation

**Figure 14:**
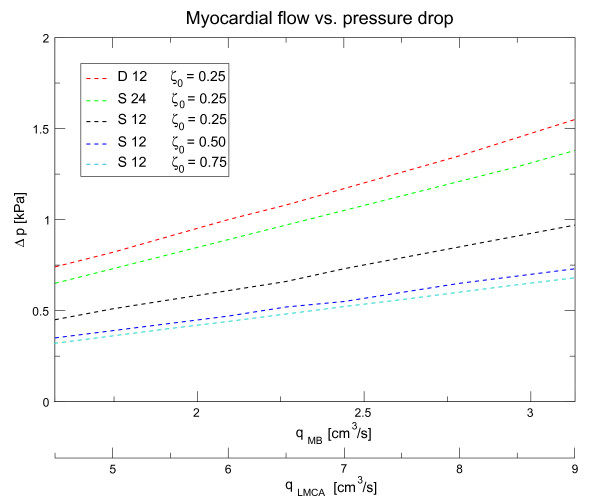
**Pressure drop vs. volume flow**. The pressure-drop flow characteristics of stenoses with different degree and extent where obtained under stationary flow conditions. We compare two single dynamic stenosis of length 12 *mm *(S12) and 24 *mm *(S24) with a double stenosis with length of 12 *mm *each (D12). Further D12 is compared for three severities *ζ*_0 _= 0.25, 0.5 and 0.75. The relations are essentially linear, extrapolation to lower inflow values leads to the origin.

The pressure-flow relations for different stenotic environments in the coronary arteries are shown in Figure 14. We have applied a stationary flow rate *q*_*LMCA *_at the entrance of the LMCA in the range between 4.5 *cm*^3^*/s *and 9 *cm*^3^*/s*. The resultant mean flow in the myocardial bridge, *q*_*MB *_was between 1.57 *cm*^3^*/s *and 3.13 *cm*^3^*/s*. The pressure-flow relations in this range are essentially linear and extrapolate to the origin at *q*_*LMCA *_= 0 and Δ*p *= 0. This is consistent with the assumption of long waves in short tubes, where the pressure-drop is linearily dependent on the flow. The single deformation with length 12 mm is denoted by (S 12) and with length 24 mm by (S 24). We further show a double deformation with length 12 mm (D 12). In general it is seen that the pressure drop increases with severity and length of the deformation, however the difference between S 24 and D 12, which have essentially the same deformation length, result from inlet and outlet effects of the double environment.

#### Influence of wall velocity

**Figure 15:**
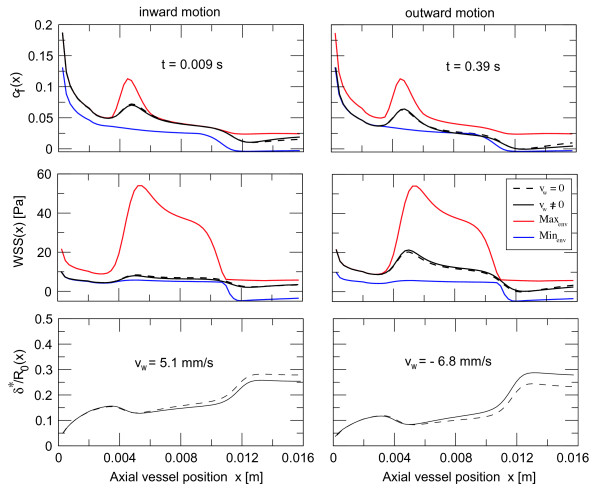
**Influence of the wall velocity on boundary layer thickness, friction coefficient and wall shear stress**. The bottom figure shows the normalised thickness of the boundary layer during in- (left) and outward motion (right) of the wall in the dynamic configuration presented in the third Additional file. Compared to zero wall velocity the boundary layer thickness in systole (*ν*_*w *_= 5.1 *mm/s*) is increased in the entrance region of the deformation while it is decreased in the outlet region, in diastole (*ν*_*w *_= -6.8 *mm/s*) the situation is vice versa. The influence on viscous friction and wall shear stress is small and thus the difference in pressure loss over the deformation compared to *ν*_*w *_= 0 is small (*δp *≈ 1% of Δ*p*).

In contrast to fixed stenoses, the velocity of the wall *ν*_*w *_≠ 0 in a dynamic environment, i.e. positive during compression and negative while the vessel is relaxing. At the bottom of Figure 15 we have shown the thickness of the boundary layer during inward (left) and outward motion (right) of the wall compared to fixed deformations. The situation depicts two different states where the influence is close to its maximum. Compared to fixed deformations (*ν*_*w *_= 0) the boundary layer thickness in systole is increased in the entrance region of the deformation, while it is decreased in the outlet region, and the situation is vice versa in diastole. In fact this is due to an additional pressure gradient, which causes fluid acceleration or deceleration depending on axial position in the constriction and whether the wall moves inwards or outwards. For example during inwards motion of the wall the fluid is decelerated at the entrance and accelerated at the outlet of the deformation, while the situation is opposite if the vessel wall moves outwards. Due to the symmetry of the indentation there is no additional acceleration or deceleration in the centre of the deformation. The influence of the wall velocity onto the boundary layer properties is more pronounced if V_W_/V is reasonably large, i.e. if the deformation that the axial flow experiences during passage of the constriction is comparable to the radius of the tube. The influence of wall velocity on viscous friction and wall shear stress is small and thus the difference in pressure loss over the deformation with *ν*_*w *_≠ 0 compared *ν*_*w *_= 0 is small (*δp *≈ 1% of Δ*p*).

## Discussion

The results have demonstrated that the formation and development of flow separation and reattachment in the post-stenotic region of a time dependent constriction are very complicated, especially secondary fluid motion in the systolic deceleration phase can cause situations of reverse flow. The post-stenotic flow is influenced by a number of factors, including the degree of stenosis, the flow and deformation waveform, and the geometry of the constriction. The above calculations suggest is that percent diameter stenosis alone does not adequately characterise the flow through myocardial bridges, and that geometric and physiological features such as the curvature, extent, and asymmetry of the stenosis, and the shape of the pulsatile waveform have substantial effects on the haemodynamic conditions.

However, we found that the total perfusion to the myocardium is strongly dependent on the severity and length of the muscle bridge. The mean FFR in fixed environment is generally smaller than in the dynamic case because the losses are not persistent during periods of small deformation, so that the pressure distal the bridge recovers during this time span. Consequently the pressure drop and flow reduction across fixed stenoses are more pronounced than in dynamic environments.

As previously noted [8] found that the pressure proximal to the myocardial bridge was higher than the aortic pressure, and concluded that the disturbance of blood flow and high wall stress proximal to the myocardial bridge was the main contributor to the development of atherosclerosis in the proximal segment. The observed wall shear stress distributions indicate that the proximal segment is more susceptible to the development of atherosclerosis, firstly because the pressure is increased there and secondly for reasons that the wall shear stress and their oscillations are maximum in the entrance region of the deformation. In contrast bridged segments are relatively spared because the wall shear stress fades towards the end of the deformation. In a series of myocardial bridges it is likely that the intima between the deformations and distal to the myocardial bridge are protected from atherosclerosis because the wall shear stress is very low and negative in separated flow regions.

## Conclusion

We have presented a method for simulation of unsteady blood flow in a time dependent vessel geometry using an integral boundary layer method. The strong interaction of the viscous boundary layer and the inviscid core flow proposed by Veldman [58] models the pressure and the extent of the separation region by assuming Falkner-Skan flow profiles. The equations were modified to the flow situation under consideration. Numerical simulations were performed for idealised stenosis geometries with a time dependent, smooth wall contour, but with a physiologically realistic coronary artery flow waveform. The predicted values of fractional flow reserve in dynamic lesions agree well with the clinical findings in [38], however, further quantification in more defined geometries is required.

Regarding the wall shear stresses and the development of atherosclerosis the findings are consistent with [1], where the intima beneath the bridge is protected from atherosclerosis, and the proximal segment is more susceptible to the development of atherosclerotic lesions.

Besides the advantage of computational time taken for the simulation, the choice of parameters, such as location, length and severity of the lesion are easily determined by coronary angiography. Due to the assumptions made in the boundary layer model, the approximation fails for the prediction of reverse flow and flow where the boundary layers merge, i.e. fully developed flow. Under these aspects severe deformations cannot be calculated, because the boundary layers merge in the deformation. Further, the length of the computational domain is restricted by the entrance length, which depends on Reynolds number. And finally the pulsatile frequency and the frequency of wall motion has to be sufficiently low (a few *Hz*), so that the approximation of quasi-stationary evolution of the boundary layers is satisfied.

We believe that the parameters and equations in this article are detailed enough to describe the physiological consequences also in a clinical setting, however, this remains to be confirmed by in vivo studies. The functional consequence, especially for severe systolic compression, is consistent with clinical findings published in the literature [1-3,6,8,38,51], where myocardial bridging is found to be responsible for myocardial ischaemia. The comparison of our findings with the published data from patient studies in [38,51] supports a potential clinical relevance of our simulation.

## Authors' contributions

SB developed the boundary layer model, carried out the simulations and drafted the manuscript. SM drafted the section on the clinical situation. AT participated in model development in its design and coordination. All authors read and approved the final manuscript.

## Supplementary Material

Additional file 1 **Animation of the left descending coronary artery assuming Hagen-Poiseuille flow**. The animation shows the pressure in the main segments of the left coronary arterial tree during one pulsatile cycle. The unit of the colour bar is *kPa*, the playback speed is slower by a factor of three than realtime. Click here for file

Additional file 2 **Animation of a double myocardial bridge in the left descending coronary artery assuming Hagen-Poiseuille flow**. As in the first animation the pressure in the main segments of the left coronary arterial tree are shown over one heart cycle. A series of two myocardial bridges with length 8 *mm *and deformation *ζ*_0 _= 0.25 are located in the mid LAD. The pressure drop is underestimated by the assumption of Hagen-Poiseuille flow, consequently perfusion to the myocardium remains nearly unaffected. The unit of the colour bar is *kPa*, the playback speed is slower by a factor of three than realtime. Click here for file

Additional file 3 **Animation of a double myocardial bridge in the left descending coronary artery using the proposed integral boundary layer method**. The animation shows the pressure in the main segments of the left coronary arterial tree over the pulsatile cycle. A series of two myocardial bridges with length 8 *mm *and deformation *ζ*_0 _= 0.25 are located in the mid LAD. It is seen that the pressure drop at the second deformation is dominant and that distal segments are less perfused. In contrast the pressure proximal to the deformation and in the left circumflex is increased [1,8]. The unit of the colour bar is *kPa*, the playback speed is slower by a factor of three than realtime. Click here for file
